# Gleanings from Our Exchanges

**Published:** 1872-04-15

**Authors:** 


### Gleanings from Our Exchanges.

Chronic enlargement of prostate gland has been much benefited by a suppository composed of one scruple of iodoform to one ounce of cocoa butter.

A Sovereign Anti-Bilious Pill.—We transcribe (by permission) from the prescription book of one who has been actively engaged in practice for the last thirty years, a formula for an anti-bilious pill, which, we feel confident, will find favor with all disposed to adopt it. As will be seen, besides containing all the components of the "Cook pill"—a pill of established popularity in the South—its also embodies other important adjuvants. The gamboge and podophyllin give increased action and augmented hepatic tendency, whilst the capsicum and extract hyosciamus impart to the whole a fine corrective and anti-drastic quality. By substituting pure castile soap for the calomel, we also have a pill that will effect all that is meant by a "Vegetable Anti-Bilious Pill:"

| | | |
|---|---|---|
| ℞ Calomel<br>Rhubarb<br>Aloes | aa. | dr. j. |
| Capsicum<br>Gamboge<br>Podophyllin,<br>Ext. Hyosciamus | aa. | gr. xij.—M. |

Make mass with Ar. Syr. Rhubarb, and divide into 22 pills. Dose from 2 to 4 for adults, as a prompt and efficient anti-bilious evacuant. —*Georgia Medical Companion.*

Dimness of Vision Following the Use of Hydrate of Chloral.—E. Burke Haywood, M.D., Raleigh, N. C. (*Rich. and Louis. Med. Jour.*), writes that he gave hydrate of chloral, in twenty-grain doses, for two weeks, to an old gentleman complaining of buzzing in the head, pulsation in the epigastrium, and soreness around the waist. At the expiration of two weeks the patient complained of dimness of vision, which, rapidly increasing, he suspected that this remedy caused it, and discontinued its use, when his vision improved, and gradually became as good as it was before using the hydrate of chloral.—*Ibid.*

Cure for Corns.—Bathe the feet well in warm water, then with a sharp instrument pare off as much of the corn as can be done without pain or causing it to bleed, and dress once a day with the following salve:

| | |
|---|---|
| ℞ Black Oxide of Copper, | gr. xv. |
| Lard, | oz. ss.—M. |

—*Ibid.*

Perchloride of Iron in Post-Partem Hemorrhage.—Dr. J. T. Johnson (*National Med. Journal*), says:

"In the Dublin Practice of Midwifery, the bold advice of Dr. Robert Barnes, of London, is followed in cases of *post-partem* hemorrhage, in the injection into the uterus of a solution of the perchloride of iron—strength, one part to four parts of water. They inject very cold water into the uterus, taking the usual precaution not to inject air, and if not successful in controlling the hemorrhage, they then use the perchloride of iron. They are much pleased with its effect, and recommend it very strongly."—*Ibid.*

Oiling Urethra in Catheterization.—It has been recommended to inject oil into the urethra previous to attempting to pass a catheter or sound in difficult cases of stricture. Dr. C. L. Carson, in *Lancet*, recommends, in place of this, filling the catheter with oil before introduction, and holding the finger over the open end until after the introduction of the end of the instrument within the urethra, when the finger may be removed.

Recurrent Vision.—Prof. A. C. Young, of Dartmouth College, mentions a fact which, so far as we know, has not been noticed before. He states that on illuminating a room with an electric spark, any object that was looked at reappeared at short intervals for two or three times. He experimented with a black screen, on which was fastened a white cross, made of strips three or four feet long by a foot in width. He is sure that the reappearance of the object is not due to a repetition of the spark, for upon moving the screen backwards and forwards, the cross always appeared in the same place. He measured the intervals between the appearance of the object by an ingenious contrivance, and finds it to be from 0·17 to 0·30. The explanation that he gives of this phenomenon is that it is due to "*a reflection of the nervous impulse* at the nerve extremities—as if the intense impression upon the retina, after being the first time propagated to the brain, were then reflected, returned to the retina, and from the retina traveling again to the brain, renewed the sensation." He calls this phenomenon "Recurrent Vision."—*Am. Jour. Science and Arts, April.*

Incontinence of Urine in Children.—Dr. Holmes Coote says that he has relieved incontinence of urine in children by the use of creasote, in every case in which he has tried it, some six in all, and when all other remedies had failed. The form in which he administers the creasote is in the form of pill one minim, with a sufficient quantity of bread crumbs.—*Lancet.*

Gangrene Produced by Dressings of Carbolic Acid.—Dr. Tillaux reports two cases in which gangrene resulted from the application of carbolic acid to wounds.

Pathology of Consumption.—Dr. Battson, says: "I cannot agree with Niemeyer that the vegetarians in diet are more subject to consumption than the well-to-do, or better fed class. Something else than an excess of *lime* in the blood is necessary for the production of tubercle.

"Louis's fatty liver of consumptives will help us to the explanation. I regard "phthisis" as much more frequently affecting the intellectual—the large-brained, the nerve-temperament. I am guessing, as you may think; but it seems to me, as *cholesterine* is the normal waste of brain and nerve tissue (*Reporter*), so is tubercle the morbid waste, or detritus, of brain and nerve tissue. Tubercle is essentially granular, composed of aplastic or retrograde cells with nuclei enclosed. An excess of phosphorized fat, as well as an excess of lime in the blood, is absolutely essential to the production of tubercle.

"I have the very best authority for saying that the rural population of France are vegetarian in diet; and yet that population are freer from consumption than any other, except, perhaps, Italy and Spain (vegetarian) in Europe, certainly than that of Great Britain, or any part of central Europe. Our New England population are largely more subject to tuberculosis of the lungs than the population of any other part of the United States, and, although largely vegetarian, still consume more fatty diet than can be appropriated to its physiological uses. Besides, so I am told, the water used in New England for drinking purposes and cooking is *soft* and not limestone, as it ought to be."—*Philadelphia Medical and Surgical Reporter.*

Progressive Pernicious Anæmia.—In the *Medicinishe Central Zeitung*, Prof. Biemer calls attention to this invariably fatal disease. He has seen fifteen cases in five years. The patients have a hydræanæmic appearance, loss of appetite, but not of fat, anæmic sounds in the arteries, capillary hemorrhages, especially of the retina, often with disturbances of the sight, some fever, and progressive debility. The *post-mortems* showed fatty degeneration of the heart and muscles.—*Idem.*

On the Employment of Taxis in Strangulated Hernia.—Dr. Smyle, in the *British Medical Journal*, calls attention to a plan for dilating the ring in strangulated hernia, and thus facilitate the reduction without the use of the knife. His plan is to crowd the finger outside of the sac into the ring, dilating it as much as possible, and then to return the tumor if possible. "For inguinal hernia in the male the index finger is applied to the lowest part of the scrotum. This is invaginated (as in Wutzer's operation for radical cure) the finger being passed behind the testicle and cord up to the external ring. The hernial tumor is then pressed downwards over the finger towards the back of the hand so as to make the structures in the ring tense, and consequently smaller. The invaginated finger is then forced firmly upwards and outwards in the direction of the internal ring. As soon as the finger is firmly grasped, the hand should be turned slightly toward the middle line. Considerable force may be safely applied in this way, as all the delicate structures are behind the finger which acts mainly on the stricture. On withdrawing the finger the hernia can usually be returned." Dr. C. C. F. Gay, of Buffalo, in an article in the *Georgia Medical Companion*, calls attention to the same method, and also says that he has had more success in the reduction of hernia when the patient was in the *upright* position and *semi-prone* position, with the thighs placed upon the body.

Opium as an Antidote to Belladonna.—Dr. Peterfield Trent reports a case of poisoning by ten grains of belladonna, in which laudanum was used with success as an antidote.—*Jour. of Materia Medica.*

Chlorate of potash in ten grain doses, together with a little morphine, has been given with excellent results in dysentery.

Aconite is said to produce serious uterine irritation if continued any length of time.

Treatment of Chronic Intermittent Fever with Enonymess Atropurpureus (Wahoo).—Dr. C. Richmond states that he has been more successful in removing enlargement of the spleen in chronic intermittents with this remedy than by any other means that he has tried. His "manner of using it is in the infusion of the fresh bark of the root; two ounces of the fresh bark of the root to one part of water, poured upon it boiling hot and allowed to steep some time. Of this, from half a pint to a pint should be given each day." He "generally gives the remedy until it purges at first, then as a diuretic and tonic." Quinine can be given at the same time.—*Ind Jour. of Medicine.*

A Lusus Naturæ.—Dr. N. Hegyetter reports a case of "a living, healthy child born with a tumor on its back containing a foot, leg, and thigh; also the rudiment of a hand, and a small placenta, or what was identical with the placenta in appearance; also a portion of the surface (inner) coated with hair, and three distinct sacs, which, along with the tumors proper contained a fluid resembling the liq. amnii."—*St. Louis Med. and Surg. Journal.*

Fluid Extract of Gelsemium has been used by Dr. W. Scott Hill in doses of from ten to fifteen drops sometimes combined with bromide of potassium, with great success in irritable bladder.

Bone Felon Arrested by Congelation.—Dr. J. B. Walker reports a case in which all "the distinguishing characteristics of a felon were manifest: pain, throbbing, some tumefaction, and the nervous excitement, indicated plainly what was in advance." He placed the finger in a mixture of equal parts of salt and snow, in a tumbler. When the finger became insensible he removed it for a moment, until the sensibility began to return, when the application was renewed. This was repeated as often as the pain returned. After about two hours of the application the pain did not return, and there was no felon.—*St. Louis Medical Archives.*

Remedy for Dandruff.—A writer in the *Amer. Jour. of Pharmacy* says that a preparation of an ounce of flowers of sulphur and a quart of water be made, the clear liquid poured off after the mixture has been shaken repeatedly at intervals of several hours, and the head wet with this mixture every morning. Simple dandruff will disappear under this treatment in a short time.

It is said that phosphate of lime will benefit the vomiting of pregnancy.

Heartburn.—A writer in the *Pharm. Jour. and Trans.* claims that heartburn is produced by butyric acid, fermentation of food in the stomach. This fermentation will be stopped by the bisulphites and proposes a bisulphite of magnesia as a remedy; magnesia having no injurious effect on the stomach as lime, soda and potash have.

Poisoning by External Application of Remedies.—The London *Lancet* (Sept., '71) reports the death of a child from an external application of an alcoholic solution of corrosive sublimate, 80 grains to the ounce. The patient was afflicted with tinea tonsurans; thirty hours after the solution was applied to the head salivation appeared, and death followed on the fifth day.—*Ibid.*

Treatment of Hemeralopia.—Dr. Philip Guntersdor (*Med. Chir. Centralbl.*, 1871) recommends electricity as a powerful remedy in this disease. He applies the induced current of moderate force by means of an electrode of wet sponge upon the forehead and eyes of the patient. Each sitting ought to last fifteen minutes, and a few sittings suffice.—*Doctor, March* 1.

Ligature of the External Iliac Artery.—High up on the right side the external iliac artery has recently been ligatured by Dr. R. G. Butcher, of St. Patrick Dun's Hospital, Dublin. Six days after operation the case is reported as progressing favorably. The operation was conducted by Dr. Butcher in nine minutes, and the necessity for its performance owing to the presence of a large inguinal aneurism which not only filled the groin, but also extended above Poupart's ligament.—*Ibid.*

Precocious Development.—Flugel describes in the *Bayr. artzl. Intell.-Blatt.*, the case of a female child who died of diarrhœa at the age of five and a-half years, having attained the height of five feet. The incisor teeth all appeared when she was six months old, and at nine months she had all the molars. At a year and a-half of age she menstruated, and, especially in her later years, the periods were tolerably regular. The external genitals, excepting the absence of hair, were well developed; the breasts were full, and the pelvis roomy. The condition of the internal genitalia was not ascertained. As regards her intellect, she did not appear to be in advance of other children of her age, although she had begun to speak when six months old.—*British Med. Journ.*

EXPERIMENTS are related which appear to prove that strychnia and morphia are antidotal to each other.

DECAISNE ON THE TREATMENT OF DELIRIUM TREMENS.—Decaisne (*Journ. Off. de la Repub. Franc.*, 1871) has made comparative experiments on the different modes of treating delirium tremens. He treated five patients with opium, and four with digitalis. The symptoms of excitement disappeared under opium on an average in five days, and with other remedies in six days. Seeing the similarity of results obtained with the different remedies, Decaisne desired to try expectant treatment, and put eight patients under this treatment; these became calm in about a similar number of days. As a consequence of these experiments, he proposes expectation as a treatment for delirium tremens, with the sole observation that all alcoholic fluids (wine, etc.) be left off, and tepid baths made use of. —*Ibid.*

A VALUABLE MEDICAL LIBRARY FOR SALE. —The valuable library of the late Professor George C. Blackman, M.D., of Cincinnati, containing upwards of three thousand volumes of medical works, besides many pamphlets, journals and papers, is now in the hands of Mr. Robert Clarke, of Cincinnati, for public sale. We agree with the editors of *American Practitioner*, that the library should be owned by some public institution or association, similar to the New York Medical Library and Journal Association.

THE UVULA.—Dr. Noble Smith condemns, in the *British Medical Journal*, the practice of snipping the uvula, and advocates its complete removal in cases where any operative procedure is called for. He relates two cases simulating consumption, which were at once cured by the removal of the elongated uvula; and says that, in mere snipping the organ grows again, and no good results. On the other hand, Sir G. D. Gibb argues in the *Lancet*, that the uvula has important functions in deglutition and vocalisation, and that its true muscular end does not often become elongated, but only the membrane, and perhaps adipose tissue; consequently, that snipping this part and leaving the muscular fibers intact is quite sufficient, and that no inconvenience arises from this practice.—*Ibid.*

DR. SAGE'S CATARRH REMEDY.—Having had occasion of late to dissolve the contents of a bottle of this great remedy in a pint of water, for a customer, its appearance induced an investigation. It is of a rather dark green color, sandy to touch, of a camphorous smell and saline taste, dissolving with facility in water, with a green, slightly opalescent color, which slowly settles to the bottom, leaving a clear yellowish solution. Twelve grains were retained, dissolved in an ounce of distilled water and one half tested with solution of nitrate of silver; an instantaneous white curdy precipitate was formed, indicating muriatic acid. Nitric acid did not change this precipitate, but upon adding a few drops of aqua ammonia it was re-dissolved. Solution of carbonate of soda produced no change in the original mixture, and a glass rod wet with the latter and held into an alcohol flame turned it yellow, characteristic of sodium. Want of time prevented the identification of the coloring matter, as well as of the odoriferous ingredient. Fifty cents is rather a high price for less than half an ounce of table salt. —*Pharmacist.*

TRANSFUSION.—It appears to some that the difficulties and dangers of this operation have been exaggerated, a position that is certainly in consonance with the results of injecting saline solutions into the veins. The *Gaz. Med. Ital. Prov. Venet.* published a case of gastric hemorrhage in a woman sixty-three years of age, who being in *articulo mortis*, was restored by the injection of one and a half ounces of defibrinated blood taken from the arm of her son. In seven days she was discharged cured from the hospital. It is stated that the warming of the blood, or the syringe, and any preparation of the vein more than is necessary for ordinary bleeding, are all useless precautions. Even the mixture of air with the blood is innocuous.

Commenting on this, the *Medical Comos* adds that the addition of such diffusible stimulants as ammonia to the blood to be injected not only serve to preserve its fluidity, but also to directly excite the heart and general circulation.

In the *Centralblatt* some cases of transfusion have been published by Juergensen, and extensively quoted. In a case of gastric ulcer, complicated with pleurisy, in which the patient was reduced to the last extremity, the operation was without benefit. Two other patients recovered, one a case of phosphorus poisoning, in which there was much hemorrhage; the other a case of asphyxia from carbonic acid, in which the benefit was most marked, and for which accident Juergensen looks upon transfusion as an important remedy.—*Ibid.*

INCONTINENCE OF URINE IN THE AGED—Dr. Schmidt recommends one drop of the tincture of iodine every two hours for this trouble. He has found marked relief follow its use.—*New York Med. Journ.*

